# Impact of Guideline-Directed Statin Prescriptions on Cardiovascular Outcomes by Race in a Real-World Primary Prevention Cohort

**DOI:** 10.1016/j.jacadv.2024.101231

**Published:** 2024-09-10

**Authors:** Eli J. Hay, Jianhui Zhu, Floyd W. Thoma, Oscar C. Marroquin, Pallavi Muluk, Malamo E. Countouris, Anson J. Smith, Gul J. Saeed, Mahmoud Al Rifai, Amber E. Johnson, Anum Saeed, Suresh R. Mulukutla

**Affiliations:** a University of Pittsburgh Medical Center (UPMC) Department of Medicine, Division of Cardiology, Pittsburgh, Pennsylvania, USA; b UPMC Heart and Vascular Institute, Department of Medicine, Division of Cardiology, Pittsburgh, Pennsylvania, USA; c Roger Williams Medical Center, Department of Internal Medicine, Providence, Rhode Island, USA; d Houston Methodist Academic Institute, DeBakey Heart & Vascular Center, Houston, Texas, USA; e University of Chicago Medicine, Department of Medicine and Section of Cardiology, Chicago, Illinois, USA

**Keywords:** ASCVD, guidelines, prevention, race, statins

## Abstract

**Background:**

Data on real-world statin prescription in large, private health care networks and impacts on primary prevention of atherosclerotic cardiovascular disease (ASCVD) outcomes across race are scarce.

**Objectives:**

The purpose of this study was to investigate the impact of statin prescription on ASCVD outcomes within and across race in a large, nongovernmental health care system.

**Methods:**

Statin prescription in Black and White patients without ASCVD was evaluated (2013-2019). Guideline-directed statin intensity was defined as at least “moderate” for intermediate and high-risk patients. Statin prescription at index and ASCVD outcomes at follow-up (myocardial infarction/revascularization, stroke, mortality) were assessed via electronic health care records using International Classification of Diseases-9 and 10 codes. Cox regression models, adjusted for CVD risk factors, were used to calculate HRs for association between statin prescription and incident ASCVD events across race.

**Results:**

Among 270,079 patients, 7.6% (n = 20,477) and 92.4% (n = 249,602) identified as Black and White, respectively. Significantly fewer Black patients were prescribed statin therapy than White patients (13.6% vs 19.0%; *P* < 0.001). At a mean follow-up of 6 years, patients with “no statin” prescription vs guideline-directed statin intensity showed increased ASCVD in Black patients (HR: 1.40 [95% CI: 1.05-1.86]), and White patients (HR: 1.32 [95% CI: 1.21-1.45]; *P* < 0.05) and all-cause mortality. Intermediate and high-risk Black patients faced a 17% higher risk of mortality compared to White patients. However, the interaction between race and statin prescription was not a significant predictor of incident ASCVD events.

**Conclusions:**

Statins remain underprescribed. Although Black patients received proportionally less statin prescription than White patients, this was not associated with higher risk of mortality in Black patients.

Statin therapy is well-established in primary prevention of atherosclerotic cardiovascular disease (ASCVD) events.^1^ Despite being a cost-efficient first-line therapy, prescription of statins in real-world settings is suboptimal, especially among Black patients.^2^, ^3^, ^4^ Additionally, Black patients are often disproportionately burdened by more frequent and severe ASCVD.^4^ Thus, the opportunity to promote equity in health outcomes in the United States persists.

Assessing patient risk of ASCVD and eligibility for statin therapy is key for primary prevention in vulnerable populations. In the United States, the American College of Cardiology/American Heart Association (ACC/AHA) guidelines are preferred for screening and identification of patients who are at risk for ASCVD events.^5^ The 2013 ACC/AHA guidelines specifically were the first to recommend calculating patient 10-year risk for ASCVD using the ASCVD Risk Estimator.^6^ These validated tools for risk estimation are useful for rapid decision-making in the prevention and management of cardiovascular disease. Despite these efforts, statin prescription in the real-world lags behind guideline recommendations and remains suboptimal.^7^

Unfortunately, Black patients in the United States have been observed to face an increased risk of all-cause mortality compared to White patients.^8^, ^9^, ^10^ Broadly, individual racism, social determinants of health, and institutional racism are all known to influence cardiovascular disease outcomes related to racial discrimination.^8^ The disparity in mortality between Black and White patients has even been reiterated in smaller analyses that adjust for markers of socioeconomic status (SES) such as neighborhood SES, income, education, and health insurance.^9^ This troublingly emphasizes the need to address structural and systemic drivers of these disparities. Given the role of statin therapy in ASCVD prevention, continued assessment of how statin prescription in Black and White patients translates to real-world outcomes could provide useful insight into where highest yield efforts can be made.

Evidence has shown that failure to adhere to guideline recommendations in eligible populations carries real-world hazard.^7^ Compared to patients who are prescribed the guideline-directed statin intensity (GDSI), patients with no statin prescription have a greater risk of ASCVD and mortality.^7^ Our study aims to assess the real-world prescription of GDSI in Black and White patients within a large health care network and evaluate if the current prescription of statins can be implicated in differences of ASCVD outcomes within and across race.

## Methods

The design and rationale of our initial investigation into statin prescription has previously been described in extensive detail.^7^ Our current analysis is a similarly structured retrospective cohort study. This study has been approved by the University of Pittsburgh PittPRO Institutional Review Board (IRB Study #18120143) and the Quality Review Committee (QRC #1281).

### Cohort derivation

Within the University of Pittsburgh Medical Center (UPMC) health system, data were gathered from 35 hospitals and approximately 400 outpatient clinics. Patients aged 20 to 79 with at least 2 UPMC health care interactions at a UPMC facility between January 2013 and December 2017 with at least one lipid result within 180 days of the first health care system interaction (index date) were included (Supplemental Figure 1). As this study focused on primary prevention of ASCVD, patients were excluded if they had a baseline history of cardiovascular disease, defined by the presence of International Classification of Diseases-9th Revision (ICD-9) and/or -10th Revision (ICD-10) code for any of the following: coronary artery disease (CAD, including myocardial infraction, angina, and revascularization, and congestive heart failure) stroke or cerebrovascular disease, transient ischemic attack, peripheral vascular disease, or rhabdomyolysis.^7^ Patients identified as receiving hospice care or care within a skilled nursing facility were also excluded.

### Covariates and pooled cohort equation-derived risk

Baseline characteristics of patients were defined using data within 180 days of their index date, as described above. These included sociodemographic data (age, sex, and race), as well as relevant social and clinical history such as smoking status, body mass index, heart rate, prescription of statin, antihypertensives, or aspirin, low-density lipoprotein cholesterol (LDL-C) level, high-density lipoprotein cholesterol (HDL-C) level, diagnosis of diabetes as well as Elixhauser Comorbidity Index scores.^11^ Patient race classification was self-identified. All data were assessed within the 180-day window after the index date. The cohort was divided into one of the following pooled cohort equation-derived 10-year risk categories: low risk (<5%), borderline risk (5%-7.4%), intermediate risk (7.5%-19.9%), and high risk (≥20%). GDSI was defined per the ACC/AHA cholesterol guidelines of 2018.^1^

The appropriate GDSI for a given patient was assessed at the index date and remained static throughout follow-up. In patients identified as intermediate risk or high risk for ASCVD events, this was defined as moderate or high intensity statin therapy per the ACC/AHA cholesterol guidelines. Any statin therapy that was of the lower than indicated intensity per guidelines was described as “less than GDSI.” High intensity statin therapy was defined as active prescription of atorvastatin 40 and 80 mg, rosuvastatin 20 and 40 mg. Moderate intensity: atorvastatin 10 and 20 mg, rosuvastatin 5 and 10 mg, simvastatin 20 and 40 mg, pravastatin 40 and 80 mg, lovastatin 40 and 80 mg. Low intensity: simvastatin 10 mg, pravastatin 10 and 20 mg, lovastatin 20 mg, fluvastatin 20 and 40 mg.

### Outcomes

The primary outcomes assessed were: 1) incident hospitalization due to CAD event including myocardial infarction and revascularization, stroke (transient ischemic attacks and/or ischemic stroke) events; 2) ASCVD outcomes of ischemic stroke, myocardial infarction, and revascularization; and 3) all-cause mortality, per each risk category. All outcomes were surveyed and included as defined per the ICD-9 and ICD-10 coding in electronic health records. Mortality was assessed using the United States Social Security Index. The interaction between race and statin prescription as it pertains to the measured ASCVD outcomes and all-cause mortality was also assessed for significance.

### Statistical analysis

Baseline descriptive statistics were initially analyzed to summarize the data and detect outliers as well as missing values. These are presented as mean and standard deviation for continuous variables or frequencies and proportions for categorical variables. Missing data for baseline descriptive measurements were uncommon, and if present replaced by the simple mean imputation for the variable across ASCVD risk groups. Sensitivity analysis performed comparing imputed data with complete-case analysis did not introduce substantial bias.

The difference of mean between race across ASCVD groups was individually assessed using the *t*-test. The chi-square test was used to compare the distribution difference of categorical variables between races. Cox proportional hazards regression models were used to compare the HRs of primary outcomes among each risk category and between patients with a level of statin usage at GDSI versus those with a level less than GDSI and/or those with no statin prescription before the first event. The proportional hazard assumption in Cox model was assessed based on the scaled Schoenfeld residuals and no significant violation was found. GDSI did not vary with time and classification of patients was based on statin status before each measured outcome. HRs were adjusted for age, sex, HDL-C, and LDL-C, systolic blood pressure, heart rate, current smoker status, history of diabetes, and hypertension treatment. This was done within and between Black and White patients. Statistical significance was set at α = 0.05. All tests of statistical significance were 2-tailed.

## Results

### Baseline demographics and statin prescription

Of 2,348,822 patients who received care at UPMC facilities with a first visit from January 2013 to December 2017, a total of 270,079 patients met the eligibility criteria. Of this, 249,602 patients identified as White, and 20,477 patients identified as Black (Central Illustration, Table 1). Among White patients, 14,338 (57.4%) were categorized as low risk, 26,177 (10.5%) as borderline, 56,371 (22.6%) as intermediate risk, and 23,667 (9.5%) as high risk. Among Black patients, 10,369 (50.6%) were categorized as low risk, 2,193 (10.7%) as borderline, 5,443 (26.6%) as intermediate risk, and 2,472 (12.1%) as high risk. Data pertaining to patients who identified as a race or ethnicity other than Black or White were determined to lack power sufficient to draw strong conclusions from.

**Central Illustration:**
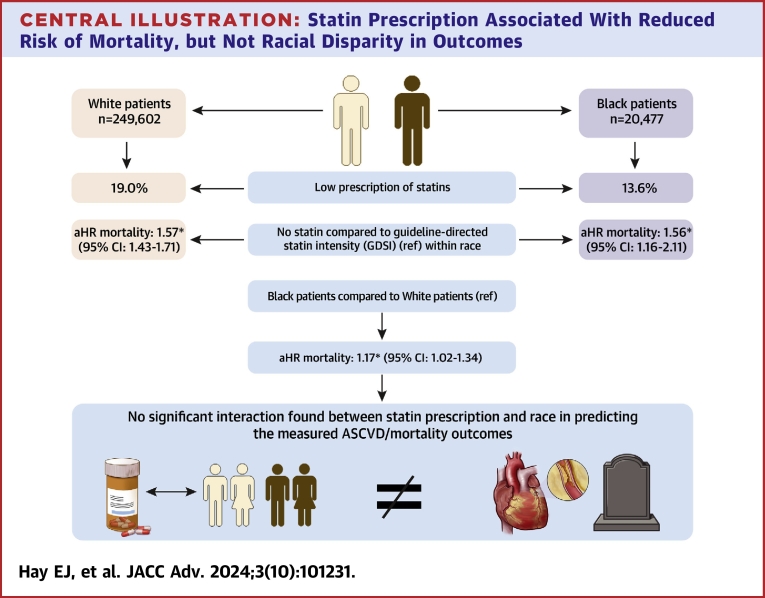
**Statin Prescription Associated With Reduced Risk of Mortality, but Not Racial Disparity in Outcomes** Data are presented as N, %, or adjusted HRs (aHRs) for mortality in high-risk patients. Outcome aHRs were compared within race between patients with no statin and guideline-directed statin intensity (GDSI), and directly across race between White and Black patients. High-risk patients were defined per the 10-year ASCVD risk categories with a risk ≥20%. High-risk GDSI was moderate or high intensity. Statin intensity and HR adjustments for cardiovascular risk factors are as defined in Methods. ∗*P* for trend <0.05. ASCVD = atherosclerotic cardiovascular disease.

**Table 1:** Baseline Characteristics by ASCVD Risk

	Total			Low Risk		Borderline Risk		Intermediate Risk		High Risk	
	White (N = 249,602)	Black (N = 20,477)	*P* Value	White (n = 14,338)	Black (n = 10,369)	White (n = 26,177)	Black (n = 2,193)	White (n = 56,371)	Black (n = 5,443)	White (n = 23,667)	Black (n = 2,472)
Age, y	50.7 ± 14.1	47.2 ± 13.7	<0.001	42.8 ± 11.1	38.1 ± 10.0	55.1 ± 8.94	50.5 ± 8.57	60.7 ± 9.12	56.3 ± 9.60	69.3 ± 8.92	62.2 ± 9.46
Age ≥75 y	3.0%	1.6%	<0.001	0.0%	0.0% ^NA^	0.0%	0.0% ^NA^	1.7%	2.1%	28.2%	9.1%
Male	43.6%	40.2%	<0.001	34.3%	28.7%	47.6%	50.8%	58.1%	53.1%	61.2%	51.0%
Female	56.4%	59.8%	<0.001	65.7%	71.3%	52.4%	49.3%	41.9%	46.9%	38.8%	49.0%
Current smoking	20.0%	31.4%	<0.001	13.1%	23.8%	29.2%	31.5%	31.0%	36.7%	25.7%	51.8%
BMI (kg/m^2^)	30.3 ± 7.1	32.5 ± 8.0	<0.001	29.8 ± 7.4	32.7 ± 8.5	31.0 ± 7.0	32.4 ± 7.7	31.0 ± 6.7	32.3 ± 7.6	31.1 ± 6.3	32.0 ± 7.4
Systolic BP (mm Hg)	127 ± 15.8	130 ± 18.0	<0.001	122 ± 13.6	122 ± 13.3	128 ± 14.5	131 ± 15.0	132 ± 15.6	137 ± 17.2	140 ± 17.8	148 ± 20.0
Heart rate (beats/min)	76.8 ± 11.6	78.9 ± 12.1	<0.001	76.9 ± 11.5	78.7 ± 11.8	77.2 ± 11.5	78.9 ± 11.6	76.8 ± 11.7	79.0 ± 12.3	75.9 ± 11.9	79.3 ± 12.9
Statin prescription	19.0%	13.6%	<0.001	9.0%	3.1%	23.4%	12.3%	31.7%	22.4%	44.4%	39.5%
Ezetimibe	0.9%	1.0%	0.486	1.00%	0.8% ^NS^	0.9%	1.1% ^NS^	0.9%	1.3%	0.90%	1.0% ^NS^
PCSK9 inhibitor	9 (0.0%)	2 (0.0%)	0.184	6 (0.0%)	1 (0.0%) ^NS^	1 (0.0%)	0 (0.0%) ^NS^	2 (0.0%)	1 (0.0%) ^NS^	0 (0.0%)	0 (0.0%) ^NA^
Total cholesterol (mg/dL)	195 ± 36.7	188 ± 35.9	<0.001	192 ± 34.5	182 ± 32.9	203 ± 37.6	192 ± 36.6	201 ± 38.7	193 ± 37.5	192 ± 40.1	194 ± 40.4 ^NS^
HTN Rx	24.8%	32.0%	<0.001	12.2%	11.4%	28.4%	32.9%	39.9%	51.1%	60.8%	75.3%
ASA	16.5%	13.6%	<0.001	6.9%	3.8%	19.3%	11.9%	28.4%	21.9%	43.0%	37.9%
HDL-C (mg/dL)	52.4 ± 15.5	53.5 ± 15.3	<0.001	55.1 ± 15.5	55.1 ± 15.0 ^NS^	50.6 ± 14.9	53.1 ± 15.5	48.8 ± 14.6	52.1 ± 15.1	47.0 ± 14.6	50.3 ± 15.8
LDL-C (mg/dL)	115 ± 32.0	111 ± 32.5	<0.001	112 ± 30.2	108 ± 30.2	122 ± 32.8	115 ± 32.7	120 ± 33.9	116 ± 34.1	112 ± 34.6	115 ± 35.6
LDL-C ≥190 (mg/dL)	2.1%	2.1%	0.910	1.4%	1.3% ^NS^	3.1%	2.5% ^NS^	3.4%	3.0% ^NS^	2.8%	3.8%
Diabetes diagnosis	10.0%	15.0%	<0.001	3.2%	4.4%	8.1%	8.1% ^NS^	14.9%	19.2%	41.0%	56.2%
Elixhauser index	0.31 ± 3.6	−0.14 ± 3.9	<0.001	−0.04 ± 3.3	−0.69 ± 3.4	0.31 ± 3.7	0.06 ± 3.9	0.70 ± 3.9	0.36 ± 4.2	1.46 ± 4.2	0.92 ± 4.6

Values are mean ± SD, %, or n (%).

*P* < 0.05 for all other comparisons between race in each ASCVD risk group.

ASA = aspirin; ASCVD = atherosclerotic cardiovascular disease; BMI = body mass index; BP = blood pressure; HDL-C = high-density lipoprotein cholesterol; HTN Rx = antihypertensive medication; LDL-C = low-density lipoprotein cholesterol; NS = not significant; NA = not applicable; PCSK9 = proprotein convertase subtilisin/kexin type 9.

On average, Black patients in this study were younger, more likely to be female, had elevated body mass index, and higher proportions of diabetes diagnosis compared to White patients. White and Black patients had the highest discordance of age in the high-risk category, with White patients being approximately 7 years older on average. Black patients, on average, were found to have higher HDL-C levels and LDL-C levels than White patients (all *P* < 0.001). Similar proportions of Black and White patients had LDL-C levels greater than or equal to 190 mg/dL (*P* = 0.910). Compared to White patients, Black patients were given significantly fewer prescriptions for statin therapy and aspirin therapy but received proportionally more prescriptions for hypertension management (Central Illustration and Table 1). Statin prescription was most discordant between race for patients at borderline risk and intermediate risk (12.3% vs 23.4% and 22.4% vs 31.6%). This difference in statin prescription decreased for those at high risk (39.5% vs 44.4%). Of note, approximately 1.4%-3.9% had a missing LDL-C value and 2.2%-2.9% of patients had missing body mass index values.

### Comparison of event frequencies between black and white patients

Stroke was the least common outcome in both races across the borderline, intermediate, and high-risk categories (Table 2). The frequency of each ASCVD outcome increased gradually as the risk level increased. Nominally, the majority of incident ASCVD events and mortality occurred in patients at intermediate and high risk for both races. The proportion of patients with each of the observed outcomes within a given risk category was highest in those at high risk.

**Table 2:** Frequency of Outcome by 10-Year ASCVD Risk and Race

	Borderline Risk		Intermediate Risk		High Risk		Total	
	White (n = 26,177)	Black (n = 2,193)	White (n = 56,371)	Black (n = 5,443)	White (n = 23,667)	Black (n = 2,472)	White (N = 106,215)	Black (N = 10,108)
CAD	486 (1.9%)	38 (1.7%)	2024 (2.6%)	174 (3.2%)	1,682 (7.1%)	155 (6.3%)	4,192 (3.9%)	367 (3.6%)
Stroke	196 (0.8%)	27 (1.2%)^a^	785 (1.4%)	112 (2.1%)^b^	767 (3.2%)	126 (5.1%)^b^	1748 (1.6%)	265 (2.6%)^b^
ASCVD	661 (2.5%)	62 (2.8%)	2,684 (4.8%)	268 (4.9%)	2,294 (9.7%)	259 (10.5%)	5,638 (5.3%)	589 (5.8%)^a^
Mortality	652 (2.5%)	44 (2.0%)	2,347 (4.2%)	244 (4.5%)	2,285 (9.7%)	194 (7.9%)^b^	5,284 (5.0%)	482 (4.8%)

Values are n (%). Data are presented as nominal incidence of CAD (MI and revascularization), stroke (ischemic), ASCVD outcomes (CAD and ischemic stroke), and mortality across 10-year risk categories. The 10-year ASCVD risk categories were defined as “borderline” (5%-7.4%), “intermediate” (7.5%-19.9%), and “high” risk (≥20%). Data for “low-risk” patients (<5%) were excluded.

CAD = coronary artery disease; other abbreviation as in Table 1.

a *P* < 0.05.

b *P* < 0.01 between White and Black patients.

### Adjusted hrs for black and white patients for events across ASCVD risk categories

At a median follow-up of 6 years, both Black and White patients with no statin prescription at their index date were at higher risk of defined ASCVD outcomes when compared to those with a prescription at GDSI (Table 3, Supplemental Table 1). This was most pronounced for both Black and White patients at high risk (Table 3) (adjusted HR [aHR]_ASCVD-Black_: 1.50 [95% CI: 1.15-1.96], *P* < 0.01; aHR_ASCVD-White_: 1.32 [95% CI: 1.21-1.44], *P* < 0.05). This significance persisted in the same manner for the outcome of all-cause mortality as well (Central Illustration, Table 3) (aHR_Mortality-Black_: 1.56 [95% CI: 1.16-2.11], *P* < 0.05; aHR_Mortality-White_: 1.57 [95% CI: 1.43-1.71], *P* < 0.05).

**Table 3:** Association of Adverse ASCVD Outcomes With Statin Prescription per 10-Year ASCVD Risk Categories Within Race

	Black Patients							
	Borderline Risk (n = 2,193)		Intermediate Risk (n = 5,443)			High Risk (n = 2,472)		
Incident Event	Any Statin (ref)	No Statin	GDSI (ref)	<GDSI	No Statin	GDSI (ref)	<GDSI	No Statin
CAD	1	1.01 (0.52-1.97)	1	1.11 (0.41-3.03)	1.30 (0.96-1.75)	1	1.01 (0.37-2.73)	1.44 (1.02-2.05)^a^
Stroke	1	2.72 (1.02-7.23)^a^	1	0.87 (0.21-3.57)	1.30 (0.89-1.90)	1	1.70 (0.69-4.20)	1.67 (1.15-2.44)^a^
ASCVD	1	1.33 (0.77-2.29)	1	1.08 (0.47-2.44)	1.33 (1.05-1.70)^a^	1	1.36 (0.69-2.66)	1.50 (1.15-1.96)^a^
Mortality	1	0.69 (0.38-1.26)	1	0.91 (0.33-2.46)	1.74 (1.34-2.25)^b^	1	0.39 (0.10-1.58)	1.56 (1.16-2.11)^a^

	White Patients							
	Borderline Risk (n = 26,177)		Intermediate Risk (n = 56,371)			High Risk (n = 23,667)		
Incident Event	Any Statin (ref)	No Statin	GDSI (ref)	<GDSI	No Statin	GDSI (ref)	<GDSI	No Statin
CAD	1	1.01 (0.83-1.21)	1	1.03 (0.79-1.35)	1.22 (1.12-1.34)^b^	1	0.93 (0.71-1.24)	1.22 (1.10-1.35)^b^
Stroke	1	1.34 (0.99-1.81)	1	1.09 (0.70-1.69)	1.54 (1.33-1.78)^b^	1	0.84 (0.53-1.34)	1.62 (1.39-1.88)^b^
ASCVD	1	1.09 (0.92-1.28)	1	1.03 (0.82-1.31)	1.29 (1.20-1.40)^b^	1	0.91 (0.71-1.16)	1.32 (1.21-1.44)^b^
Mortality	1	1.78 (1.50-2.10)^b^	1	1.17 (0.91-1.51)	1.65 (1.51-1.79)^b^	1	1.22 (0.98-1.52)	1.57 (1.43-1.71)^b^

Data are presented as HRs and CIs of CAD (MI and revascularization), stroke (ischemic), ASCVD outcomes (CAD and ischemic stroke), and mortality across 10-year risk categories. The 10-year ASCVD risk categories were defined as “low risk” (<5%), “borderline” (5%-7.4%), “intermediate” (7.5%-19.9%), and “high” risk (≥20%). GDSI for each risk category is defined per ACC/AHA cholesterol guidelines of 2013 and 2018, and models of association were adjusted for the pooled cohort equation variables; both described in Methods.

ACC = American College of Cardiology; AHA = American Heart Association; GDSI = guideline-directed statin intensity; MI = myocardial infarction; other abbreviations as in Tables 1 and 2.

a *P* for trend <0.05.

b *P* for trend <0.001.

For Black patients, those who were at intermediate risk of ASCVD with no statin prescription at their index date were associated with a significantly higher risk for defined ASCVD outcomes (aHR_ASCVD_: 1.33 [95% CI: 1.05-1.70], *P* < 0.05) as well as mortality (aHR_Mortality_: 1.74 [95% CI: 1.34-2.25]) compared to similar risk Black patients with prescription of GDSI. This significance did not maintain when individually assessing the MI and stroke outcomes. Conversely, White patients at intermediate risk with no statin prescription at their index date faced a significantly higher risk of defined ASCVD outcomes and mortality (aHR_ASCVD_: 1.29 [95% CI: 1.20-1.40]; aHR_Mortality_: 1.65 [95% CI: 1.51-1.79]) compared to similar risk White patients with prescription of GDSI. However, unlike for Black patients, the association did extend to higher risk of MI and stroke outcomes individually for intermediate risk White patients (aHR_CAD_: 1.22 [95% CI: 1.12-1.34], *P* < 0.001; aHR_Stroke_: 1.54 [95% CI: 1.33-1.78], *P* < 0.001). Thus, while the adjusted HR for MI and stroke was higher for the “no statin” groups in both intermediate risk White and Black patients, statistical significance was not met in the latter.

### Adjusted HRs of events between black and white patients

There was no significant difference in risk of defined ASCVD outcomes when comparing Black and White patients at intermediate and high risk (Central Illustration, Table 4). However, Black patients showed a significantly higher risk of mortality (aHR_Mortality_: 1.17 [95% CI: 1.01-1.36], *P* < 0.05; aHR_Mortality_: 1.17 [95% CI: 1.02-1.34], *P* < 0.05) in the intermediate and high-risk groups, respectively. The interaction between statin prescription and race was not a significant predictor for any of the outcomes assessed for those at intermediate and high risk (Supplemental Table 2).

**Table 4:** Association of Adverse ASCVD Outcomes Between Race per 10-Year ASCVD Risk Categories

Incident Event	Intermediate Risk		High Risk	
	White Patients (ref)	Black Patients	White Patients (ref)	Black Patients
CAD	1	1.10 (0.94-1.29)	1	0.95 (0.80-1.13)
Stroke	1	1.06 (0.70-1.62)	1	0.94 (0.63-1.42)
ASCVD	1	1.25 (1.10-1.42)^b^	1	1.13 (0.99-1.30)
Mortality	1	1.17 (1.01-1.36)^a^	1	1.17 (1.02-1.34)^a^

Data are presented as HRs and CIs of CAD (MI and revascularization), stroke (ischemic), ASCVD outcomes (CAD and ischemic stroke), and mortality across 10-year risk categories. The 10-year ASCVD risk categories were defined as “intermediate” (7.5%-19.9%) and “high” risk (≥20%). Models of association adjusted for the pooled cohort equation variables and covariates including statin prescription as described in Methods.

Abbreviations as in Tables 1 and 2.

a *P* for trend <0.05.

b *P* for trend <0.001.

## Discussion

In this retrospective analysis of statin prescription within a large, nongovernmental, real-world cohort of patients in the greater Western Pennsylvania area, we sought to categorize how statins are currently prescribed, the subsequent effect on risk of ASCVD, and if the prescription of these medications can be associated with health disparities in ASCVD outcomes across race. We found that statin prescription continues to be associated with decreased risk of ASCVD events and all-cause mortality. Black and White patients at high risk that were not prescribed any statin on index were at approximately 56% higher risk of mortality compared to similar race patients that were prescribed GDSI. There also continues to be significant underprescription of statin therapy in the community at large, as less than half of all patients at high risk were prescribed a statin on index. Overall, Black patients were prescribed proportionally less statin therapy than White patients and this effect was most profound in patients at borderline and intermediate risk. We also found continued overall disparities in outcomes between Black and White patients: Black patients classified as intermediate and high risk had a 17% increase in risk of all-cause mortality compared to similarly stratified White patients. Risk for incident myocardial infarction and stroke was similar when compared across race for these groups. However, the interaction term between race and statin prescription was not a significant predictor of the outcomes assessed. Thus our findings suggest that the beneficial effects of statin prescription on mortality were similar in both races.

The data support the known protective association between statin therapy and reduced risk of ASCVD events.^1^^,^^5^^,^^7^ This is most obvious in patients whose 10-year ASCVD risk profiles are “high.” At the median follow-up of 6 years in this cohort, both Black and White patient sample showed that patients who were designated with “no statin” prescribed at their index date were at an increased risk of defined ASCVD outcomes when compared to patients within race who were prescribed with the GDSI. This is in line with the robust literature advocating the efficacious, low-risk profile of statins.^12^

The real-world implementation of guidelines for statin prescription in primary prevention of ASCVD remains a crucial area for improvement. In our data, less than half of patients at high risk had no evidence of a statin prescription on index. While concerning, there is optimism for improvement as patients not receiving a statin despite eligibility report that a leading reason for doing so was never being counseled by their clinician on starting therapy.^13^ It is therefore possible that noninitiation of statin therapy accounts for a sizeable portion of the underprescription observed in our model. Fortunately, patients with no history of statin prescription are generally amenable to trialing statin therapy and given that statins are relatively affordable and well-tolerated, our results emphasize an opportunity for realistic improvement in the primary prevention of ASCVD.^13^ Excluding low-risk patients in our study, a plurality of patients were classified as intermediate risk which troublingly yielded some of the highest disparities in statin prescription rates; approximately a 9% difference between Black and White patients. This intermediate risk designation may pose a unique challenge for providers in reliably assessing patient ASCVD risk, therefore improving provider recognition of patient ASCVD risk and statin eligibility may be a fruitful avenue of action. It will likely become of greater importance to refine risk assessment processes for patients who would benefit from intervention and offering of statin prescription, especially as machine learning, artificial intelligence, and precision medicine continue to gain inertia.^14^

Examining across race, we found worse mortality outcomes and inequitable statin prescriptions for Black patients when compared to White. However, we did not find a significant interaction between statin prescription, mortality, and race in our current analysis. Precise explanations for the findings at hand are beyond the scope of our analysis; however, we suspect the stress and hazard of sustained structural inequities in society to be primarily responsible for the mortality outcomes we observed.^15^^,^^16^ In the context of statin utilization, factors such as age, race, sex, poverty, and health insurance rates have previously been seen to have compounding negative impacts on use prevalence of these medications.^17^ Regrettably, such effects are potentially intensified in context of differences across race in perceptions of cardiovascular risk and statin safety.^18^ Beyond statin utilization, markers of SES have been theorized and observed to have profoundly negative associations with all-cause and cardiovascular mortality.^8^^,^^9^ Per the Pittsburgh Inequities across Gender and Race (PIGR) Report, Pittsburgh as a case-study is known to suffer significantly from racial disparities across these determinants.^16^ Black and White residents of Pittsburgh differ significantly in poverty rates, income, employment rate and type, and education level with Black residents bearing the unfavorable side of comparison within each.^16^ The overall mortality rate, and rate of death by cardiovascular disease for Black residents in Pittsburgh was higher than that for Black residents in 98% of similarly sized cities.^16^ Although our model does not specifically account for social determinants of health, our results suggest that statin prescription is not currently implicated in the worse mortality outcomes for Black patients. As such, known socioeconomic disparities are likely to be exercising significant influence over the mortality outcomes we observed in our current analysis.

A uniquely worrying discrepancy in the demographic makeup of our study is that approximately 7.5% of our study sample was Black, whereas the general proportion of Black residents in Pittsburgh is documented to be about 22%.^16^ While our sample is partially derived from facilities outside of the city limits of Pittsburgh, most major facilities are located within city limits. Although our data are sourced from a broad variety of hospitals and clinics in Western Pennsylvania, this discrepancy does limit the overall generalizability of our data as this level of incongruency may be driven by region-specific factors. However, this amplifies the concern for the health disparities across race that we have categorized as it appears that our data are not fully capturing a sizeable proportion of Black residents for reasons most likely related to access to care.

Unfortunately, our data are likely to be yet another reflection of continued inequities across race in our geographic area. The health care industry is known to have had a significant role in the promulgation of these inequities through many unethical and abusive acts toward communities of color.^19^ Substantive efforts pursuant of equity in health care are wide-ranging; with areas of potential impact including but not limited to promotion of diversity, equity, and inclusion programs in medical training, as well as advocating for structural programs to empower community members.^20^ Efforts such as our study are important in the evaluation of current manifestations of structural racism and the progress that needs to be made. In whichever manner, we emphasize addressing the structural nature of this subject before seeking pharmaceutical interventions as the solution for a societal problem that is far more complex.

### Study limitations

Our study has notable limitations as it relates to the analysis of driving forces behind the increased risk of mortality we observed in Black patients. Inability to capture relevant social determinants of health in the current analysis limits the depth of exploration regarding the socio-politico-economic context of our data. Therefore, our model alone cannot be used to further explain the driving forces specifically within the general population. However, other studies have sought to understand how SES varies across race in Pittsburgh as well as how SES markers may impact all-cause and cardiovascular mortality.^9^^,^^16^ We do not suspect that our study is unique in this regard. Other limitations inherent to the retrospective collection of data from existing electronic health records include lack of data such as prescription filling, and an incomplete ability to assess the diagnosis of peripheral arterial disease, as well as coronary calcium scores, a known modulator of ASCVD risk. Uniquely impactful to this exploration is the role of regional influences on patient demographic makeup that have been observed to bias investigations into health inequities across race similar to our current effort.^2^ Additionally, our data set was comprised by a majority White patients, with only 7.5% of the sample being Black. As discussed above, this is not in line with population data indicating that Black residents compose approximately 22% of Pittsburgh’s demographic make-up.^16^ Despite this, we were still able to detect significant differences in the risk of mortality between race in intermediate to high-risk patients. Our data still provide utility for local and neighboring constituents in assessing the real-world performance of the greater Western Pennsylvania area. Despite a large overall sample, our results cannot be extrapolated to the entire U.S. population, nor can they indicate causality given the retrospective nature.

## Conclusions

Our data from a large, real-world health care system show that statins remain underused in both Black and White patients. Real-world prescription of statins is associated with lowered risk of adverse ASCVD outcomes. Black patients from our sample were overall observed with an increased risk of mortality compared to White patients. Despite this, statin prescription did not interact significantly with race to impact prediction of the ASCVD outcomes measured.

Perspectives **COMPETENCY IN MEDICAL KNOWLEDGE:** Statin therapy remains a useful agent in the real-world reduction of adverse ASCVD outcomes. In the absence of a significant interaction between statin prescription and race in predicting adverse ASCVD events, the data suggest looking beyond pharmaceutical intervention with statins to reduce disparities in cardiovascular disease outcomes.

**TRANSLATIONAL OUTLOOK:** The data in our study illustrate that despite overall increased risk of mortality in Black patients at high risk of ASCVD, statins did not significantly interact with race to impact this outcome. Although statins were observed to be efficacious within race for the mortality outcome, this likely indicates other culprit variables, likely to be social determinants. Further research from large-health systems will benefit from inclusion of social determinants of health in patient demographic data. Additionally, this retrospective cohort study also serves as an example of in-house data analysis within a large, private health care system. As the roots of artificial intelligence in medicine continue to grow, efforts such as ours may serve as a nidus for further research in this area developing geography specific clinical tools.

## Funding support and author disclosures

The authors have reported that they have no relationships relevant to the contents of this paper to disclose.
