# Supplementary material for: Impact of Guideline-Directed Statin Prescriptions on Cardiovascular Outcomes by Race in a Real-World Primary Prevention Cohort
Source: JACC Adv. 2024 Sep 10;3(10):101231. doi: 10.1016/j.jacadv.2024.101231 (PMC11414661; doi:10.1016/j.jacadv.2024.101231)
Supplement: Supplementary materials [file mmc1.docx]

**Supplemental Figure 1:** Selection of Cohort Population from University of Pittsburgh Medical Center Records


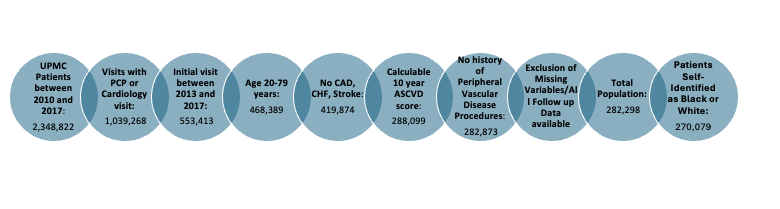


**Abbreviations:** UPMC; University of Pittsburgh Medical Center, PCP; primary care physician (including outpatient internal medicine, general internal medicine and family medicine), ASCVD; atherosclerotic cardiovascular disease; CAD; coronary artery disease, CHF; congestive heart failure

**Supplemental Table 1. Overall Association of Adverse ASCVD Outcomes with Statin Prescription per 10-y ASCVD Risk Categories**

| **ASCVD** | **Borderline** | | **Intermediate** | | | **High** | | |
| --- | --- | --- | --- | --- | --- | --- | --- | --- |
| **Event** | Any statin  (ref) | No statin | GDSI  (ref) | < GDSI | No statin | GDSI  (ref) | < GDSI | No statin |
| **CAD** | 1 | 1.01  (0.84-1.21) | 1 | 1.04  (0.80-1.34) | 1.23  (1.13-1.34) † | 1 | 0.94  (0.72-1.23) | 1.23  (1.11-1.36) † |
| **Stroke** | 1 | 1.43  (1.08-1.90) * | 1 | 1.06  (0.70-1.62) | 1.51  (1.33-1.73) † | 1 | 0.94  (0.63-1.42) | 1.63  (1.41-1.87) † |
| **ASCVD** | 1 | 1.10  (0.94-1.29) | 1 | 1.03  (0.83-1.30) | 1.30  (1.20-1.40) † | 1 | 0.95  (0.75-1.19) | 1.33  (1.23-1.45) † |
| **Mortality** | 1 | 1.68  (1.42-1.98) † | 1 | 1.16  (0.91-1.47) | 1.65  (1.52-1.80) † | 1 | 1.16  (0.93-1.47) | 1.56  (1.44-1.70) † |

Data are presented as hazard ratios and confidence intervals of CAD (MI and revascularization), stroke (ischemic), ASCVD outcomes (CAD and ischemic stroke) and mortality across 10-year risk categories. The 10-year ASCVD risk categories were defined as “borderline” (5%-7.4%), “intermediate” (7.5-19.9%) and “high” risk (≥20%). GDSI for each risk category are defined per ACC/AHA Cholesterol guidelines of 2013 and 2018, and models of association were adjusted for the pooled cohort equation variables; both described in *Methods*.

**Abbreviations:** ACC: American College of Cardiology; AHA: American Heart Association; ASCVD: atherosclerotic cardiovascular disease; CAD: coronary artery disease; GDSI: Guideline directed statin intensity; MI: myocardial infarction

*** p-value for trend <0.05**

† **p-value for trend <0.001**

**Supplemental Table 2**: Interaction Between Patient Race and Statin Prescription in Predicting Risk of Adverse ASCVD Outcomes

|  | **Borderline risk** | **Intermediate risk** | **High risk** |
| --- | --- | --- | --- |
| **Event** | **p-value** | **p-value** | **p-value** |
| **CAD** | 0.98 | 0.54 | 0.31 |
| **Stroke** | 0.173 | 0.708 | 0.399 |
| **ASCVD** | 0.482 | 0.973 | 0.399 |
| **Mortality** | 0.003* | 0.786 | 0.287 |

Data presented are p-values derived from Cox Regression Models assessing significance of interaction between race and statin prescription on risk of adverse ASCVD outcomes including CAD (MI and revascularization), stroke (ischemic), ASCVD (composite CAD and ischemic stroke) events and mortality across 10-year risk categories. The 10-year ASCVD risk categories were defined as “intermediate” (7.5-19.9%) and “high” risk (≥20%). Models of association were adjusted for the pooled cohort equation variables.

**Abbreviations:** ASCVD: atherosclerotic cardiovascular disease; CAD: coronary artery disease

* p-value for trend <0.05
